# Subclinical Cushing's Disease with High-Molecular-Weight Forms of Adrenocorticotropic Hormone Production

**DOI:** 10.1155/2024/8721614

**Published:** 2024-03-25

**Authors:** Takahiko Inukai, Nozomi Harai, Yukie Nakagawa, Tadatsugu Hosokawa, Airi Antoku, Yuko Muroi, Masakazu Ogiwara, Kyoichiro Tsuchiya

**Affiliations:** ^1^Department of Diabetes and Endocrinology, University of Yamanashi Hospital, Yamanashi, Japan; ^2^Department of Neurosurgery, University of Yamanashi Hospital, Yamanashi, Japan

## Abstract

Production of the high-molecular-weight forms of adrenocorticotropic hormone (big-ACTH) has been reported in a small number of ectopic ACTH syndrome and ACTH-producing pituitary macroadenoma. However, perioperative changes in big-ACTH in patients with subclinical Cushing's disease have not been reported. A 63-year-old woman presented 25 × 20 × 20-mm-sized macroadenoma in the pituitary gland. Her early morning plasma ACTH and cortisol levels were 111 pg/mL and 11.6 *μ*g/dL, respectively. Cushingoid features and diurnal variation in plasma cortisol levels were not observed. The patient's urinary free cortisol (UFC) was 59.3 *μ*g/day. The corticotropin-releasing hormone (CRH) test showed that plasma ACTH levels were 1.5 times higher than the preload value. The overnight dexamethasone suppression test (DST) showed that the plasma cortisol level was not suppressed by 0.5 mg of dexamethasone (DEX) but was suppressed by 8 mg of DEX. Inferior pyramidal sinus sampling was consistent with Cushing's disease. Taken together, the patient was clinically diagnosed with subclinical Cushing's disease caused by an ACTH-producing pituitary adenoma. Endoscopic transsphenoidal adenomectomy was performed. In the postoperative CRH test, plasma ACTH levels showed six-fold increase. The postoperative DST showed cortisol suppression at 0.5 mg of DEX. The UFC levels decreased to 35.1 *μ*g/day. Pituitary contrast-enhanced MRI revealed no residual tumor, and plasma ACTH and cortisol levels remained within normal ranges. Gel filtration of preoperative and postoperative plasma ACTH was performed, and a high molecular weight fraction of ACTH was detected, which markedly decreased postoperatively. The absence of Cushingoid features and the lack of significant cortisol hypersecretion in this case were thought to be due in part to big-ACTH, which has low bioactivity. By careful evaluation of laboratory and clinical findings, we identified it as a big-ACTH-producing adenoma. This is the first report of a case in which the big-ACTH transition was observed perioperative and is a valuable case.

## 1. Introduction

Subclinical Cushing's disease is generally defined as hypercortisolism resulting from the hypersecretion of adrenocorticotropic hormone (ACTH) from the pituitary adenoma without overt Cushingoid features [1]. Compared with adrenal subclinical Cushing's syndrome, cases of subclinical Cushing's disease have been reported less to date [1–4].

The high-molecular-weight forms of ACTH (big-ACTH) are precursors of ACTH (1–39) that are produced during the formation of ACTH from proopiomelanocortin (POMC) expressed in the anterior pituitary gland. Cushing's syndrome associated with the big-ACTH production has been reported in ectopic ACTH syndrome [5, 6] and ACTH-producing pituitary macroadenoma [3, 4, 7]. A case of subclinical Cushing's disease with pituitary adenoma in which a big-ACTH was produced has been reported [8]. However, perioperative changes in the big-ACTH in patients with subclinical Cushing's disease have not been documented.

Here, we presented a case of subclinical Cushing's disease without an increase in basal plasma cortisol levels, despite high plasma ACTH levels. Gel chromatography detected big-ACTH and ACTH (1–39) in the preoperative plasma sample, which diminished after surgery for the pituitary tumor.

## 2. Case Presentation

A 63-year-old Japanese woman presented to an ophthalmologist 2 months prior to presentation with intermittent diplopia. The patient was referred to the University of Yamanashi Hospital following the suspicion of oculomotor nerve palsy. She was not a smoker but was an occasional drinker. The patient had no family history of endocrine disorders. She was taking amlodipine (5 mg) for hypertension and rosuvastatin (2.5 mg) for dyslipidemia. Her height, weight, body mass index, blood pressure, and pulse rate were 154 cm, 67 kg, 28.3 kg/m^2^, 130/84 mmHg, and 70 beats/min, respectively. Cushingoid features, including moon face, rosacea, hypertrichosis, buffalo hump, red skin striations, muscle atrophy, muscle weakness, or skin thinning, were not observed. Cranial nerve examination revealed no ocular motor deficits.

**Figure 1:**
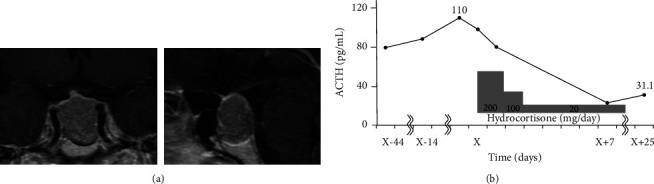
Clinical course after the appearance of diplopia. (a) Contrast-enhanced magnetic resonance imaging of pituitary tumors. (b) The solid line indicates the ACTH level. ACTH, adrenocorticotropic hormone.

The laboratory data from the early morning after admission are presented in Table 1. Plasma ACTH levels were measured using electrochemiluminescence immunoassay (ECLusys ACTH™; Roche Diagnostics K.K., Tokyo, Japan), and the patient's ACTH and plasma cortisol levels were 111 pg/mL and 11.6 *μ*g/dL, respectively. The patient's dehydroepiandrosterone sulfate level was within the normal range for age and sex. No electrolyte or blood cell abnormalities were observed. The patient's urinary free cortisol (UFC) level was also within the normal range (Table 2). Pituitary contrast-enhanced magnetic resonance imaging (MRI) revealed a 25 × 20 × 20-mm-sized macroadenoma with a poor contrast effect from the sella turcica to the suprasellar region, and the normal pituitary gland was compressed cephalad to the lesion (Figure 1(a)). The patient's optic chiasm deviated to the right, and visual field examination revealed typical bitemporal hemianopsia (data not shown). Contrast-enhanced computed tomography of the cervical-to-pelvic region showed no tumors or enlargement of the adrenal gland.

**Figure 2:**
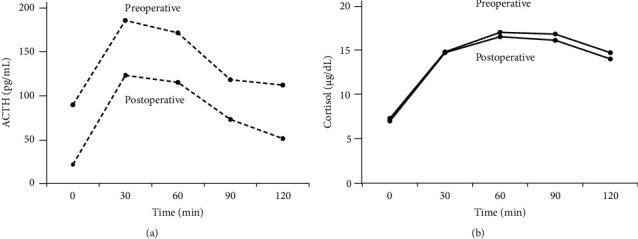
The results of preoperative and postoperative CRH loading tests. The dotted line on the (a) represents the ACTH level. The solid line on the (b) illustrates the cortisol levels. ACTH, adrenocorticotropic hormone.

Diurnal fluctuations indicated an unsuppressed plasma cortisol level (5 *μ*g/dL) at 23 : 00 (Table 3). The corticotropin-releasing hormone (CRH) (100 *μ*g) test showed that the plasma ACTH level was 1.5 times higher than the preload value (Figure 2). The overnight dexamethasone (DEX) suppression test (DST) showed that the plasma cortisol level was not suppressed by 0.5 mg of DEX but was suppressed by 8 mg of DEX (Table 4). Inferior pyramidal sinus sampling was performed to rule out an ectopic ACTH syndrome. Sampling showed the plasma ACTH level central/peripheral ratios of >2 on the right side at the baseline and >3 bilaterally at the apex after CRH stimulation (Table 5). Bromocriptine (2.5 mg) decreased the plasma ACTH levels to 55.7 pg/mL at the lowest level, but octreotide acetate (50 *μ*g) did not decrease ACTH levels for 24 h (data not shown). Taken together, the patient was clinically diagnosed with subclinical Cushing's disease caused by an ACTH-producing pituitary adenoma.

**Figure 3:**
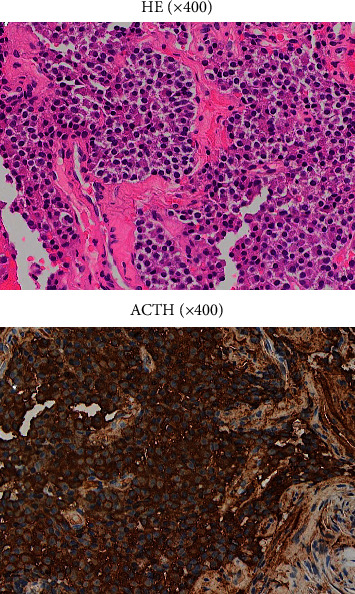
Immunostaining of excised pituitary adenomas. The upper figure represents hematoxylin-eosin staining, and the lower figure represents ACTH staining. H&E, hematoxylin-eosin staining; ACTH, adrenocorticotropic hormone.

Four months after referral to our hospital, endoscopic transsphenoidal adenomectomy was performed. The tumor specimen showed monotonous cell proliferation with eosinophilic spores and was ACTH-positive on immunostaining (Figure 3). Growth hormone, thyroid-stimulating hormone, luteinizing hormone, follicle-stimulating hormone, and prolactin were negative. Ki-67 was 2–3%. At 168 h after surgery, plasma ACTH levels decreased to 23 pg/mL. Although hydrocortisone was perioperatively administered for postoperative ACTH suppression, plasma ACTH levels gradually decreased to normal ranges after surgery without suppression. Hydrocortisone replacement was terminated 10 days after surgery (Figure 1(b)).

In the postoperative CRH test (100 *μ*g), plasma ACTH levels showed a six-fold increase (Figure 2). Plasma cortisol levels increased during the preoperative CRH test (Figure 2). The postoperative DST showed cortisol suppression at 0.5 mg of DEX (Table 4). The UFC level decreased to 35.1 *μ*g/day (Table 2). Twenty-eight weeks after surgery, pituitary contrast-enhanced MRI revealed no residual tumor, and plasma ACTH and cortisol levels remained within normal ranges.

**Figure 4:**
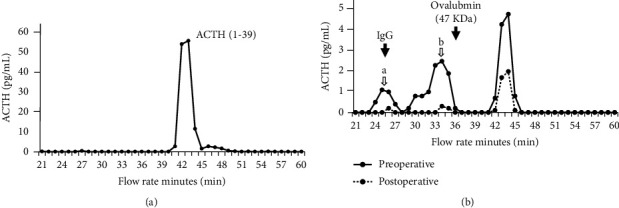
Pre- and postoperative ACTH sample fraction assay after gel filtration. The above figure represents ACTH (1–39), and the figure below represents pre- and postoperative ACTH samples fractionated by gel filtration, separated by time, and measured. The solid lines represent preoperative data, and the dotted lines represent postoperative data. (a) 25-min values; (b) 34-min values. ACTH, adrenocorticotropic hormone.

Gel filtration of pre- and postoperative plasma ACTH was performed. After protein removal by hydrochloric acid treatment, centrifugal filtering was performed and the supernatant was applied. The solute was fractionated 20–60 min after injection, and ACTH concentration in each fraction was determined by an AIA-PACK CL ACTH reagent (Tosoh Corporation, Tokyo, Japan) using AIA-CL automated immunoassay analyzers (Tosoh Corporation, Tokyo, Japan). As a control, a fraction assay in which the ACTH (1–39) standard was treated by gel filtration in the same manner was also performed. ACTH measurement revealed that plasma before surgery showed that in addition to the ACTH (1–39) peak observed at approximately 44 min, high molecular weight fractions were detected at approximately 25 and 34 min (Figure 4). These high molecular weight peaks decreased after surgery.

## 3. Discussion and Conclusions

The present case report described a patient with pituitary adenoma that produced big-ACTH, resulting in subclinical Cushing's disease. Although big-ACTH production associated with ectopic ACTH syndrome [5, 6] and Cushing's disease [3, 4, 7] have been reported, big-ACTH-producing pituitary adenoma without overt Cushingoid features, as in the present case, have rarely been reported [8]. In addition, this is the first report demonstrating the decreases in the plasma big-ACTH in subclinical Cushing's disease before and after surgery.

A 39 amino acid residue ACTH is synthesized from a larger precursor molecule, termed POMC, via specific processing by prohormone convertase (PC) 1/3 in a tissue-specific manner [9]. In the normal anterior pituitary, POMC is processed to *β*-lipotropin and pro-ACTH, which is further cleaved into an N-terminal peptide containing *γ*-melanocyte-stimulating hormone (MSH) (N-POC), joining peptide, and ACTH (1–39). In contrast, aberrant processing of POMC and/or enhanced processing of ACTH (1–39) into *α*-MSH and corticotropin-like intermediate lobe peptide by nonpituitary tumors leads to an increased proportion of circulating big-ACTH and ACTH-related fragments in patients with ectopic ACTH syndrome [10]. The activity of PC 1/3 is decreased in the big-ACTH-producing ectopic ACTH syndrome and Cushing's disease [10]. Thus, although it was not assessed, PC 1/3 expression/activity in the pituitary tumors in the present case might have been low.

In the present case, although plasma cortisol levels were unsuppressed at midnight and by 0.5 mg of DEX, they were consistently within normal ranges. As the big-ACTH are less biologically active than ACTH (1–39) [10], the effect of the pituitary tumor-derived big-ACTH on cortisol production in the present case was likely weaker than that of ACTH (1–39). Moreover, relatively low plasma cortisol levels may have contributed to the subclinical phenotype in the present case.

In this case, inferior pyramidal sinus sampling was performed to rule out an ectopic ACTH syndrome. Several reports have shown big-ACTH production from nonpituitary organs/tissues [5, 6]. It has been reported that ectopic ACTH syndrome is atypically associated with cortisol suppression in a high-dose dexamethasone suppression test [11]. Then, ACTH-dependent autonomous cortisol production associated with a pituitary adenoma does not always indicate Cushing's diseases. Indeed, the Japanese guideline of Cushing's syndrome/disease recommends inferior pyramidal sinus sampling even to cases of ACTH-dependent autonomous cortisol production associated with a pituitary tumor.

Complete surgical resection of pituitary adenomas is associated with postoperative hypocortisolism and suppressed CRH response because of long-standing corticotroph suppression in the normal gland [12]. Consequently, low postoperative cortisol and ACTH levels have been used as markers of remission from Cushing's disease. Some reports have proposed that postoperative hypocortisolemia (<5 *µ*g/dL), reduced plasma ACTH level (<10 pg/mL), or suppressed CRH response can accurately identify patients in remission from Cushing's disease after surgery [12–15]. However, in the present case, reduced plasma ACTH level and suppressed CRH response were not observed after surgery. Given that the big-ACTH, which are likely to be less biologically active than ACTH (1–39), were produced by the tumor in the present case, the effect of corticotroph suppression in the normal gland might be atypically weak. Certainly, the present case showed the postoperative suppression of the plasma cortisol level by 0.5 mg dexamethasone, indicating resolution of autonomous ACTH-dependent cortisol production by surgery.

Although the big-ACTH were markedly reduced by surgery, they were only slightly detected postoperatively. It does not always indicate residual tumor because POMC is secreted from keratinocytes and the pituitary gland [16] and that POMC and/or pro-ACTH was detected in the plasma of normal subjects [17, 18]. Because the postoperative clinical course of Cushing's disease with the production of big-ACTH remains unknown, careful postoperative observation with biochemical and imaging tests was required in the present case.

When autonomous cortisol secretion is suspected but its plasma levels are not elevated, corticosteroid-binding globulin (CBG) deficiency should be considered. In humans, 80–90% of cortisol is bound to CBG and other proteins, and 5–10% is in the free active form [19]. When CBG is deficient, the rates of production and excretion of free cortisol relatively increase. In a case report of Cushing's syndrome with CBG deficiency, the plasma cortisol level was normal, but the UFC level was elevated [20]. The present case was not associated with an increased UFC level or conditions that decrease the CBG level (e.g., polycystic ovarian syndrome, hypoproteinemia, or septic shock), suggesting that CBG deficiency is not involved in the pathophysiology.

In summary, we report a case of subclinical Cushing's disease with pituitary adenoma in which surgery results in a marked decrease in the plasma big-ACTH. As the production of big-ACTH can result in atypical hormonal findings and changes, careful and integrated interpretation of all findings and test results, considering the pathophysiology, is required.

## Figures and Tables

**Table 1:** The laboratory data for the early morning after admission.

Biochemistry	
TP	6.3 g/dL
Alb	3.6 g/dL
T-bil	0.5 mg/dL
AST	16 U/L
ALT	12 U/L
ALP	207 U/L
*γ*-GTP	55 U/L
BUN	12.4 mg/dL
Cr	0.63 mg/dL
TG	80 mg/dL
LDL-C	217 mg/dL
HDL-C	43 mg/dL
Na	144 mEq/L
K	3.4 mEq/L
Cl	110 mEq/L
HbA1c	5.8%
BG	87 mg/dL
Hematology	
WBC	4170 *µ*L
Neutro	2130 *µ*L
Lympho	1680 *µ*L
Mono	220 *µ*L
Eosino	100 *µ*L
Baso	0 *µ*L
Hb	12.2 g/dL
RBC	392 × 10^4^ *µ*L
Hct	37.9%
Plt	16.5 × 10^4^ *µ*L
Endocrine	
ACTH	92.6 pg/mL
Cortisol	7.7 *µ*g/dL
DHEA-S	78 *µ*g/dL
PRL	12.4 ng/mL
TSH	0.893 *µ*IU/mL
Free T3	2.79 pg/mL
Free T4	1.16 ng/dL
FSH	20.2 mIU/mL
LH	10.4 mIU/mL
GH	0.53 ng/mL
IGF-1	77 ng/mL
PAC (CLEIA)	54 pg/mL
PRA	0.6 ng/mL/h
Urinary	
pH	6.0
Protein	±
Glucose	—
Occult blood	—

TP, total protein; BG, blood glucose; WBC, white blood cell; Neutro, neutrophil; Lympho, lymphocyte; Mono, monocyte; Eosino, eosinophil; Baso, basophil; Hb, hemoglobin; RBC, red blood cell; Hct, hematocrit; Plt, platelets; ACTH, adrenocorticotropic hormone; DHEA-S, dehydroepiandrosterone sulfate; PRL, prolactin; TSH, thyroid-stimulating hormone; FSH, follicle-stimulating hormone; LH, luteinizing hormone; GH, growth hormone; IGF-1, insulin-like growth factor-1; PAC, plasma aldosterone concentration; CLEIA, chemiluminescent enzyme immunoassay; PRA, plasma renin activity.

**Table 2:** Urinary free cortisol (*µ*g/day).

Pre	Post
59.3	35.1

Pre, preoperative; Post, postoperative.

**Table 3:** Preoperative diurnal variation.

	7:00	15:00	23:00
ACTH (pg/mL)	92.6	79.5	64.4
Cortisol (*µ*g/dL)	7.7	4.3	5.0

ACTH, adrenocorticotropic hormone.

**Table 4:** Overnight dexamethasone suppression test.

DEX	0.5 mg		8 mg	
	Pre	Post	Pre	Post
ACTH (pg/mL)	82.9	8.16	84.3	3.53
Cortisol (*µ*g/dL)	7.5	1.5	3.5	<1

DEX, dexamethasone; ACTH, adrenocorticotropic hormone; Pre, preoperative; Post, postoperative.

**Table 5:** Plasma ACTH levels in inferior pyramidal sinus blood sampling before and after CRH stimulation.

	Before			After		
	C	*P*	C/P ratio	C	*P*	C/P ratio
Right	1,222	81.1	15	2,000	105	20
Left	132	81.1	1.6	971	105	9.7

ACTH, adrenocorticotropic hormone; CRH, corticotropin-r; C, central; P, peripheral.

## Data Availability

The datasets used and/or analyzed during the current study are available from the corresponding author on reasonable request.
